# Evaluation of the illumigene Mycoplasma Direct DNA Amplification Assay

**DOI:** 10.1128/JCM.01930-17

**Published:** 2018-06-25

**Authors:** Neena Kanwar, Morgan A. Pence, Donna Mayne, Jeffrey Michael, Rangaraj Selvarangan

**Affiliations:** a Children's Mercy Hospital and Clinics, Kansas City, Missouri, USA; b Cook Children's Medical Center, Fort Worth, Texas, USA; c Sacred Heart Hospital, Pensacola, Florida, USA; Johns Hopkins University School of Medicine

**Keywords:** Mycoplasma pneumoniae, illumigene assay, LAMP technology

## Abstract

Mycoplasma pneumoniae is a common cause of community-acquired pneumonia. The illumigene Mycoplasma Direct (iMD) DNA amplification assay is a qualitative *in vitro* test utilizing loop-mediated isothermal amplification (LAMP) technology for the direct detection of M. pneumoniae DNA in respiratory specimens. The iMD assay does not require the preextraction of nucleic acids from specimens, which is a prerequisite step for the previously approved illumigene Mycoplasma (iM) assay. The aim of this prospective multicenter study was to evaluate the performance characteristics of the newly developed iMD assay, compared with the iM assay. Subjects with symptoms of upper respiratory illnesses suggesting M. pneumoniae infection were enrolled at three sites in the United States. Respiratory specimens were obtained using dual throat swabs. One swab was tested with the iMD assay at each enrollment site. Reference testing with the iM assay was performed by the manufacturer. Among 456 specimens tested, the iM reference method detected M. pneumoniae in 25 specimens (5.5%), while the iMD assay identified 34 specimens (7.5%) as M. pneumoniae positive. There were 10 false-positive results and 1 false-negative result with the iMD assay. The overall positive and negative agreement rates were 96.0% (95% confidence interval [CI], 80.5 to 99.3%) and 97.7% (95% CI, 95.8 to 98.7%), respectively. The overall agreement rate was determined to be 97.6% (95% CI, 95.7 to 98.6%). We conclude that the iMD test results were comparable to the iM assay results. The removal of the DNA extraction step for the iMD assay simplifies testing, saves time, and reduces the costs of detecting M. pneumoniae from throat swabs, compared to the iM assay.

## INTRODUCTION

Mycoplasma pneumoniae is a common cause of respiratory tract infections, especially in young adults and school-age children. Diagnosis of M. pneumoniae infection based on a patient's clinical presentation alone is difficult and unreliable (1). Prompt diagnosis is essential to initiate appropriate antibiotic therapy and infection control.

Several methods are utilized for M. pneumoniae detection. Culture-based methods are highly specific and sensitive but are time-consuming, and their sensitivity may vary depending on laboratory skills (2, 3). Serology-based assays (complement fixation assay, enzyme-linked immunoassay, enzyme-linked immunosorbent assay, and microparticle agglutination assay) are available. The limitations of serology-based assays include a lack of sensitivity due to a delayed antibody response to M. pneumoniae infection, a lack of response in older patients, highly prevalent background antibodies in healthy individuals, and cross-reactivity with other Mycoplasma species (2, 4, 5). Rapid antigen kits targeting specific proteins are also being utilized to diagnose M. pneumoniae. The sensitivity of rapid antigen assays has been reported to vary from 60% to 90% (2, 6, 7).

Nucleic acid amplification testing (NAAT) assays have gained popularity due to increased assay sensitivity (1, 2), which enables detection at an early stage of infection. The loop-mediated isothermal amplification (LAMP) assay is a NAAT assay that is simple, easy to use (no thermocycler is needed), and highly sensitive, with a quick turnaround time (2). The illumigene Mycoplasma (iM) assay (Meridian Bioscience, Inc., Cincinnati, OH) utilizes LAMP technology for the detection of M. pneumoniae from throat and nasopharyngeal swab specimens. The primers of this diagnostic kit target a 208-bp DNA sequence found in the intracellular protease-like protein gene of the M. pneumoniae genome (1). A byproduct of the continuous isothermal amplification reaction is magnesium pyrophosphate. This white precipitate leads to turbidity in the reaction mixture. The illumipro-10 incubator/reader monitors the changes in the absorbance characteristics. Significant changes in the absorbance of the reaction mixture indicate the presence of the target gene. A study found the sensitivity and specificity of the iM assay to be 100% and 99%, respectively, compared with the culture method (1). Another recent study found both sensitivity and specificity of the iM assay to be 100%, compared with the FilmArray respiratory panel (bioMérieux, France) (8).

The company manufacturing the iM assay recently developed an improved version, the illumigene Mycoplasma Direct (iMD) assay, which does not require a Qiagen DNA extraction step. The gene target and assay chemistry are identical for the iM and iMD assays. This multicenter clinical trial compared the clinical performance of the iMD assay with that of the iM assay, which requires nucleic acid extraction. This is the first study evaluating the performance of the iMD assay.

## MATERIALS AND METHODS

### Study design.

This prospective multicenter clinical trial was conducted at three different sites (Florida, Texas, and Missouri) across the United States. Patients with upper respiratory tract infections that might be attributed to M. pneumoniae were enrolled after informed consent was obtained. Fresh throat swab specimens from both male and female patients were collected between August 2015 and January 2016. This study was designed to evaluate the performance of the newly improved iMD assay, compared with the iM assay. The study protocols were approved by each hospital's institutional review board.

### Specimens.

Specimen inclusion criteria included (i) specimens collected from patients with symptoms of upper respiratory tract infections that might be attributed to M. pneumoniae or patients suspected of having M. pneumoniae infection, (ii) two throat swabs from each subject enrolled, one transported in nonnutrient transport medium and other in M4 medium, and (iii) written informed consent provided by the subject. Specimen exclusion criteria included (i) specimens received in the laboratory in unsatisfactory containers or conditions, (ii) multiple sets of specimens from the same patient at different office visits, (iii) specimens received in the laboratory with less than two throat swabs per subject, and (iv) specimens stored in unapproved transport medium types or combinations. Demographic data collected at enrollment included age, gender, date of symptom onset, antibiotic use in past 4 weeks, and other medication use. No other clinical or radiological findings were recorded.

A total of 456 subjects at the three different sites were included in the study. A summary of the subjects enrolled and M. pneumoniae prevalence across the three sites is provided in Table 1. Patients 3 weeks to 97 years of age with upper respiratory tract illnesses that might be attributable to M. pneumoniae were included. A dual-swab (rayon tip) collection device was used to collect one specimen per patient. One swab was stored in liquid Amies medium (catalogue no. 220105; Becton, Dickinson and Company, Franklin Lakes, NJ, USA) and the second was stored in M4 transport medium (catalogue no. 12500; Thermo Fisher Scientific; Remel Products, Lenexa, KS, USA). Specimens were stored at 2 to 8°C prior to testing. At each study site, specimens were deidentified and assigned a unique, site-specific, study identifier. Testing was performed with the iMD assay with one throat swab at each study site. Operators at each study site tested external positive- and negative-control samples every day prior to performing the iMD assay with the study samples. The second swab, in M4 medium, was shipped to the manufacturer for testing with the iM assay. The results from the two assays were compared to determine the performance of the iMD assay.

**TABLE 1:** Total subjects enrolled and M. pneumoniae prevalence across the three sites

Site no. and location	No. of subjects		M. pneumoniae prevalence (%)
	Total	Total positive	
Site 1, Florida	152	4	2.6
Site 2, Texas	49	4	8.1
Site 3, Missouri	255	26	10.2
Total	456	34	7.5

### illumigene Mycoplasma Direct assay.

The swab stored in red-capped nonnutrient liquid Amies transport medium was utilized to perform the iMD assay, at the clinical site, within 72 h after sample collection. Briefly, the swab was inserted by breaking the swab handle in the SMP Prep tube provided with the kit. The SMP Prep tube was vortex-mixed for 10 s. Five to 10 drops from the SMP Prep tube were squeezed into a 1.5-ml heat treatment tube. The heat treatment tube was heated at 95°C for 10 min, followed by 10 s of vortex-mixing. Fifty microliters of this heat-treated sample was transferred to the test chamber as well as the control chamber of the illumigene test device. The device was closed, and all air bubbles were removed by gently tapping the test device. The illumigene test device was inserted into an illumipro-10 incubator/reader for direct detection of M. pneumoniae. Results were displayed on the instrument as positive or negative at the conclusion of the run, in less than 1 hour.

### illumigene Mycoplasma assay.

Specimens stored in M4 medium were shipped (transport temperature, 2 to 8°C) to the company within 4 days after sample collection and were tested within 14 days. The Qiagen QIAamp DSP DNA minikit was utilized to extract DNA from 150 μl of the specimen. The comparator iM assay was performed by the company according to the assay package insert.

### Statistical analysis. (i) Descriptive statistics.

Overall characteristics of the subjects enrolled and the M. pneumoniae prevalence at each individual site were determined with reference to the iMD assay. M. pneumoniae prevalence was also determined according to age and gender for each of the three sites.

### (ii) Analytical statistics.

Data tables (two by two) were utilized to determine the rates of positive and negative agreement between the iM assay and the iMD assay. Analysis of the performance characteristics (positive and negative agreement rates), with 95% confidence intervals (CIs), was performed using the Vassarstats website (http://vassarstats.net/clin1.html).

## RESULTS

A total of 456 subjects with symptoms of upper respiratory tract illness, from three different sites, were enrolled in the study. The highest prevalence was observed at the Missouri site (10.2%), followed by the Texas site (8.1%) (Table 1). There was an approximately equal distribution of male and female subjects enrolled in the study (female, *n* = 230 [50.4%]; male, *n* = 226 [49.6%]) (Table 2). The median age of the patients included in the study was 5 years (range, 0.06 to 97 years). The highest M. pneumoniae prevalence was observed in the age group of 3 to 12 years (22/147 patients [15%]), compared to the rest of the study population (*P* < 0.001). The association of age with M. pneumoniae prevalence has been documented in previous studies (9–11). M. pneumoniae prevalence rates among female and male patients (7.0% and 8.0%, respectively) were found to be comparable (*P* = 0.7). Detailed age- and gender-specific prevalence rates across all sites are listed in Table 2.

**TABLE 2:** M. pneumoniae prevalence according to age and gender across all three sites, as determined with the iMD assay

Age and gender	No. of subjects					M. pneumoniae prevalence (%)
	Site 1 (Florida)	Site 2 (Texas)	Site 3 (Missouri)	Total	Total positive	
Age group						
0–1 mo	0	0	7	7	0	0.0
2 mo to 2 yr	9	12	136	157	5	3.2
3–12 yr	20	28	99	147	22	15.0
13–21 yr	16	9	13	38	5	13.2
22–65 yr	86	0	0	86	2	2.3
>65 yr	21	0	0	21	0	0.0
Gender						
Male	63	24	139	226	18	8.0
Female	89	25	116	230	16	7.0
Total	152	49	255	456	34	7.5

Among the 456 specimens tested, the iMD and iM assays detected M. pneumoniae in 34 specimens (7.5%) and 25 specimens (5.5%), respectively. There were 10 false-positive specimens and 1 false-negative specimen detected by the iMD assay. The rates of positive and negative agreement between the iMD assay and the iM assay were 96.0% and 97.7%, respectively. Discrepancy analysis was performed by repeat testing of a second aliquot of the discrepant sample with the iM assay. Four of 10 false-positive specimens were identified as positive by the iM assay after retesting with an additional frozen sample. Repeat testing of 1 false-negative specimen with the iM assay, using the original patient sample and an additional frozen sample, produced negative results in both cases.

Detailed performance parameters for all study subjects and specific parameters for the study population are presented in Table 3. No invalid runs were observed during the study. Results of standard-of-care tests for M. pneumoniae detection (an enzyme immunoassay and PCR for the first site, the iM assay for the second site, and the BioFire FilmArray respiratory panel for the third site) were available for 70 of the 456 subjects enrolled in the study, with 11 positive detections.

**TABLE 3:** Performance characteristics of the iMD assay, compared with the iM assay (with Qiagen extraction)

iMD assay result	No. of samples^*a*^		
	Positive iM assay result	Negative iM assay result	Total
Positive	24	10^*b*^	34
Negative	1^*c*^	421	422
Total	25	431	456

a Overall agreement, 97.6% (95% CI, 95.7 to 98.6%); positive agreement, 96.0% (80.5 to 99.3%); negative agreement, 97.7% (95.8 to 98.7%).

b Four of 10 samples were identified as positive by the iM assay after testing with an additional frozen swab collected from the patients for discrepancy analysis.

c Repeat testing with the iM assay, with the original patient sample and an additional frozen sample, produced negative results.

## DISCUSSION

The objective of this multisite clinical trial was to evaluate the performance characteristics of the iMD assay, compared to the iM assay. This is the first study evaluating the performance of the iMD assay.

A previous study (8) in the United States compared the iM assay, the Prodesse ProPneumo-1 assay (Hologic Gen-Probe, San Diego, CA, USA), and the Mycoplasma pneumoniae P1 LightMix kit (TIB Molbiol, Howell, NJ, USA) with the FilmArray respiratory panel (bioMérieux, France) for detection of M. pneumoniae from pediatric clinical nasopharyngeal swab specimens. All of the three commercially available NAAT assays had similar sensitivities, specificities (100%, 100%, and 96% for the iM assay, the Prodesse assay, and the LightMix kit, respectively), and hands-on times. Another study compared the iM assay with culture, using frozen respiratory specimens from adults and children for whom historic culture results were available (1). The sensitivity and specificity of the iM assay after discrepancy analysis were observed to be 100% and 99%, respectively. In our study, the overall rate of agreement between the iMD assay and the iM assay was found to be 97.6%. Given the study results, it is expected that the iMD assay will perform comparably, compared with the existing diagnostic platforms.

Current FDA-cleared sample-to-answer molecular methods for M. pneumoniae detection are the nested multiplex PCR panel by BioFire Inc. and standalone LAMP assays (iM and iMD assays) by Meridian Bioscience, Inc. The iMD assay targets the intracellular protease-like protein gene by utilizing LAMP technology (no thermocycler is needed) and provides results in less than 1 hour. By comparison, the FilmArray respiratory panel targets the *tox* gene for M. pneumoniae detection. Advantages of the iMD assay over the FilmArray respiratory panel for M. pneumoniae detection are that both instrument costs and costs per test are lower for the iMD test and the iMD assay has a higher throughput (it can process 10 specimens, compared with a single specimen, at one time), making it ideal for testing in an outpatient setting. One study evaluated the performance of the FilmArray respiratory panel for detection of M. pneumoniae versus a laboratory-developed real-time TaqMan PCR assay targeting the *p1* gene (12) and determined the positive agreement rate to be 100% (95% CI, 70.1 to 100%), which was comparable to the value of 96.0% (95% CI, 80.5 to 99.3%) for both iM and iMD assays obtained in our study. We evaluated 25 true-positive M. pneumoniae isolates, compared with 9 positive isolates utilized in the FilmArray respiratory panel study (12); this resulted in relatively more robust estimates, as indicated by the 95% CIs.

Several studies conducted in Europe have compared various commercially available NAAT assays for M. pneumoniae detection (13–15), and a summary of commercially available NAAT assays for detection of M. pneumoniae was provided in a recent minireview article (16). These NAAT assays need specialized trained personnel, expensive setups with thermal cycling amplification platforms, and a molecular biology facility. In contrast, the LAMP technology utilized in the iMD assay allows specific and continuous DNA amplification under isothermal conditions. The iMD assay is a sensitive assay that does not require an additional DNA extraction step, making it faster and more economical than other NAAT assays that require the DNA extraction step. Overall, the iMD assay is a simple, convenient, and rapid molecular assay for detection of M. pneumoniae from throat swab specimens.

## References

[1] Ratliff AE, Duffy LB, Waites KB 2014 Comparison of the *illumi*gene Mycoplasma DNA amplification assay and culture for detection of *Mycoplasma pneumoniae*. J Clin Microbiol 52:1060–1063. doi:10.1128/JCM.02913-13. 24430454 PMC3993520

[2] Parrott GL, Kinjo T, Fujita J 2016 A compendium for *Mycoplasma pneumoniae*. Front Microbiol 7:513. doi:10.3389/fmicb.2016.00513. 27148202 PMC4828434

[3] Diaz MH, Winchell JM 2016 The evolution of advanced molecular diagnostics for the detection and characterization of *Mycoplasma pneumoniae*. Front Microbiol 7:232. doi:10.3389/fmicb.2016.00232. 27014191 PMC4781879

[4] Youn Y-S, Lee K-Y 2012 *Mycoplasma pneumoniae* pneumonia in children. Korean J Pediatr 55:42–47. doi:10.3345/kjp.2012.55.2.42. 22375148 PMC3286761

[5] Waites KB, Balish MF, Atkinson TP 2008 New insights into the pathogenesis and detection of *Mycoplasma pneumoniae* infections. Future Microbiol 3:635–648. doi:10.2217/17460913.3.6.635. 19072181 PMC2633477

[6] Miyashita N, Kawai Y, Tanaka T, Akaike H, Teranishi H, Wakabayashi T, Nakano T, Ouchi K, Okimoto N 2015 Diagnostic sensitivity of a rapid antigen test for the detection of *Mycoplasma pneumoniae*: comparison with real-time PCR. J Infect Chemother 21:473–475. doi:10.1016/j.jiac.2015.02.007. 25818195

[7] Yamazaki T, Kuroki H, Itagaki T, Iwata S, Tateda K 2015 Evaluation of a rapid antigen detection kit targeting L7/L12 ribosomal protein for *Mycoplasma pneumoniae*. Kansenshogaku Zasshi 89:394–399. doi:10.11150/kansenshogakuzasshi.89.394. 26552132

[8] Chou RC, Zheng X 2016 A comparison of molecular assays for *Mycoplasma pneumoniae* in pediatric patients. Diagn Microbiol Infect Dis 85:6–8. doi:10.1016/j.diagmicrobio.2015.12.013. 26830272

[9] Chen ZR, Yan YD, Wang YQ, Zhu H, Shao XJ, Xu J, Ji W 2013 Epidemiology of community-acquired *Mycoplasma pneumoniae* respiratory tract infections among hospitalized Chinese children, including relationships with meteorological factors. Hippokratia 17:20–26. 23935339 PMC3738272

[10] Othman N, Isaacs D, Kesson A 2005 *Mycoplasma pneumoniae* infections in Australian children. J Paediatr Child Health 41:671–676. doi:10.1111/j.1440-1754.2005.00757.x. 16398873

[11] Defilippi A, Silvestri M, Tacchella A, Giacchino R, Melioli G, Di Marco E, Cirillo C, Di Pietro P, Rossi GA 2008 Epidemiology and clinical features of *Mycoplasma pneumoniae* infection in children. Respir Med 102:1762–1768. doi:10.1016/j.rmed.2008.06.022. 18703327

[12] Pierce VM, Elkan M, Leet M, McGowan KL, Hodinka RL 2012 Comparison of the Idaho Technology FilmArray system to real-time PCR for detection of respiratory pathogens in children. J Clin Microbiol 50:364–371. doi:10.1128/JCM.05996-11. 22116144 PMC3264185

[13] Touati A, Benard A, Hassen AB, Bébéar CM, Pereyre S 2009 Evaluation of five commercial real-time PCR assays for detection of *Mycoplasma pneumoniae* in respiratory tract specimens. J Clin Microbiol 47:2269–2271. doi:10.1128/JCM.00326-09. 19403761 PMC2708483

[14] Dumke R, Jacobs E 2014 Evaluation of five real-time PCR assays for detection of *Mycoplasma pneumoniae*. J Clin Microbiol 52:4078–4081. doi:10.1128/JCM.02048-14. 25210063 PMC4313229

[15] Pillet S, Lardeux M, Dina J, Grattard F, Verhoeven P, Le Goff J, Vabret A, Pozzetto B 2013 Comparative evaluation of six commercialized multiplex PCR kits for the diagnosis of respiratory infections. PLoS One 8:e72174. doi:10.1371/journal.pone.0072174. 24058410 PMC3751960

[16] Loens K, Ieven M 2016 *Mycoplasma pneumoniae*: current knowledge on nucleic acid amplification techniques and serological diagnostics. Front Microbiol 7:448. doi:10.3389/fmicb.2016.00448. 27064893 PMC4814781

